# Numerical Cognition in Bees and Other Insects

**DOI:** 10.3389/fpsyg.2013.00162

**Published:** 2013-04-18

**Authors:** Mario Pahl, Aung Si, Shaowu Zhang

**Affiliations:** ^1^BEEgroup, Biocentre, Julius-Maximilians-UniversityWürzburg, Germany; ^2^Department of Linguistics, School of Culture, History and Language, College of Asia and the Pacific, The Australian National UniversityCanberra, ACT, Australia; ^3^Centre of Excellence in Vision Science, Research School of Biology, The Australian National UniversityCanberra, ACT, Australia

**Keywords:** bees, insects, counting, learning, memory, numerical cognition, quantity discrimination

## Abstract

The ability to perceive the number of objects has been known to exist in vertebrates for a few decades, but recent behavioral investigations have demonstrated that several invertebrate species can also be placed on the continuum of numerical abilities shared with birds, mammals, and reptiles. In this review article, we present the main experimental studies that have examined the ability of insects to use numerical information. These studies have made use of a wide range of methodologies, and for this reason it is striking that a common finding is the inability of the tested animals to discriminate numerical quantities greater than four. Furthermore, the finding that bees can not only transfer learnt numerical discrimination to novel objects, but also to novel numerosities, is strongly suggestive of a true, albeit limited, ability to count. Later in the review, we evaluate the available evidence to narrow down the possible mechanisms that the animals might be using to solve the number-based experimental tasks presented to them. We conclude by suggesting avenues of further research that take into account variables such as the animals’ age and experience, as well as complementary cognitive systems such as attention and the time sense.

## Introduction

Insects are not the hardwired reflex automats they were once believed to be. Especially central place foragers like ants and bees, who venture out to provide the nest with a constant flow of resources, show impressive navigation and communication skills. The animals cover large distances in search of food and nesting material, and usually take the shortest route back home, even when the nest entrance is out of sight. Insects have evolved sophisticated ways to acquire, memorize, and act upon information collected from the environment. When navigating to a food source, honeybees employ a celestial compass (Von Frisch, 1949) and a visual odometer (Esch and Burns, 1995; Tautz et al., 2004) to measure the distance and the direction of their movement. This information is integrated to continuously update a homeward vector, so that the forager can return to the nest from any point on the foraging route in a straight line (Wehner et al., 1990). Bees memorize the most profitable flowers depending on the location (Zhang et al., 2006), and the time of day (Pahl et al., 2007). Ants navigate by a similar mechanism, albeit the distance information is acquired by integrating steps, rather than using optic flow (Wittlinger et al., 2006). “Number” is another property of visual scenes which animals can learn and use, in order to maximize foraging efficiency. Most studies on numerical competence have focused on vertebrates, but there is a growing body of evidence showing that the ability to use numerical information is not restricted to this group. For example, the use of quantity in predatory behavior has recently been demonstrated in an araneophagic spider. These communal predators base their decision about settling near a prey spider nest on the number of conspecifics already present, preferring one spider over zero, two, and three (Jackson and Nelson, 2012; Nelson and Jackson, 2012). There is evidence that 17-year periodical cicadas (*Magicicada* sp.) could be counting the seasonal cycles of trees in order to hatch after precisely 17 years, instead of using the passage of real time or degree days (Karban et al., 2000). Mealworm beetles (*Tenebrio molitor*) have been shown to discriminate between odor bouquets containing the scents of different amounts of females (Carazo et al., 2009), and to keep a running tally of the number of encountered males to inform mate-guarding decisions (Carazo et al., 2012). Most of the work on invertebrate numerical competence has focused on social insects, because they may particularly benefit from a sense of number. As central place foragers, they face more demanding navigational problems than animals without a nest to return to between foraging bouts. In this paper, we review the advances in our understanding of how and why insects use numerical information in mating strategies (Carazo et al., 2009, 2012), navigation (Chittka and Geiger, 1995; Reznikova and Ryabko, 1996; Wittlinger et al., 2006; Dacke and Srinivasan, 2008), foraging (Bar-Shai et al., 2011a,b), and visual decision-making (Leppik, 1953; Gross et al., 2009).

Gelman and Gallistel (1978) have defined a set of five criteria for true counting: one-to-one correspondence, stable order, cardinality, abstraction, and order irrelevance. Since symbolic labels are required for the items to be labeled, these criteria are difficult to apply to non-verbal animals. True counting according to Gelman and Gallistel has thus so far only been shown in Chimpanzees (Matsuzawa, 2009) and grey parrots (Pepperberg, 2006) using arabic numerals. The data on numerical competence in insects presented in this review shows different levels of sophistication, but none of the animals displayed true counting in the sense of Gelman and Gallistel. Number-related behavior in animals in which not all of the strict “true counting” criteria are fulfilled can be described as “proto-counting” (Davis and Pérusse, 1988).

## What is the Advantage of a “Sense of Number”?

Numerical competence can be useful in many ways, i.e., when selecting the best foraging and mating grounds, tracking predators, in social interactions, in parenting and preventing brood parasitism. Social insects, as central place foragers, can profit from a sense of number in navigation. A running count of landmarks can inform the navigator about its progress, or indicate when it is approaching the destination. A sequence of landmarks along a route could also be helpful in calibrating a bee’s odometer – since landmarks interact with odometric information to enhance the accuracy of navigation (Chittka et al., 1995; Srinivasan et al., 1997; Vladusich et al., 2005). There is evidence that landmarks are memorized together with a vector encoding distance and direction to the hive or the food source (Cartwright and Collett, 1983), and used to update the internal homeward vector (Collett, 1992). The number of a landmark might also be helpful when one landmark in a row has to be identified, e.g., a particular tree from a row of trees may be combined with a vector memory, or the correct entrance to the nest in an array of hives in a large apiary could be identified by its numerical order. Desert ants (*Cataglyphis fortis*) use a “step counter” to measure traveled distances, enabling them to constantly update information about the distance and direction of the nest entrance (Wittlinger et al., 2006). Bees are often flower-constant, and numerical information could be used to identify nectar-bearing flowers, along with color, shape, and scent (Leppik, 1953; Gross et al., 2009). Visual information about number may also inform foraging decisions based on the number of bees already present on a flower. Numerical regularities in flowers can be used to forage more efficiently, by avoiding revisits at already depleted nectaries (Bar-Shai et al., 2011b).

## Number in Mating Strategies

The polygynandrous mating system of the yellow mealworm beetle (*Tenebrio molitor*) leads to intense sperm competition. Male beetles have evolved several strategies to turn the odds in their favor, some of which require quantity estimation and – discrimination. In order to optimally adjust their mating behavior, male beetles need to acquire information about the number of male and female animals in the group. Carazo et al. (2009) have tested if male beetles discriminate between odor bouquets containing the odors of 1, 2, 3, and 4 female beetles in a 2 choice situation. They found that the animals discriminated between 1 vs. 4, and 1 vs. 3 females, but not between 1 vs. 2, or 2 vs. 4 females. The results suggest that males are capable of chemically discriminating between two odor sources based on the number of females contributing to the odor – if the ratio exceeds 1:2. In order to maximize his mating opportunities, the male beetle will always go for more.

In a further study, Carazo et al. (2012) investigated if male mealworm beetles adjust the amount of time spent mate-guarding in response to the amount of rival males encountered before the mating event. During mating, sperm is transferred to the female’s bursa in a spermatophore. The release of the sperm, however, occurs 7–10 min after the copulation. If the female re-mates with a second male in this time window, the second male can prevent the sperm release from the first spermatophore. Thus, a male beetle should guard its mate for a while after copulation – if the risk of spermatophore inhibition is high. To test whether male beetles can estimate the number of present males, Carazo et al. staged matings in which they varied the number of rivals the experimental male encountered before the mating event. They found that the beetles increased the time spent guarding the female in response to the amount of rival males encountered before mating. The authors conclude that the animals keep a running tally of serially encountered individuals, and use numerosity estimation to inform their mate-guarding decisions.

Numerical competence is likely to play an important role in many different species for which the assessment of sperm competition risk and intensity is vital. Shifferman (2012) argues that its role in determining males’ responses to sperm competition can expose quantity estimation to selection, and thus facilitate its evolution.

## Number in Navigation

In times of scarce resources, honeybees often fly four or more kilometers from the hive to collect pollen or nectar. On those long foraging trips, the bee’s on-board dead reckoning system constantly integrates the distance flown and the angle of movement, by measuring the optic flow over the retina (Esch and Burns, 1995; Esch et al., 2001) and the body angle relative to the solar meridian (Labhart, 1980; Rossel and Wehner, 1986). In theory, this enables the animal to know the distance and direction back to the hive from any point on her outward route (Wehner et al., 1990). On long trips, however, errors in the measurements accumulate, and the bee needs additional strategies in order to reset or calibrate its path integrator (Srinivasan et al., 1997; Cheung et al., 2007; Merkle and Wehner, 2010). One way bees achieve this is by memorizing landmarks on the route together with a vector encoding distance and direction to the hive or the food source (Cartwright and Collett, 1983), and using those landmarks to update the internal homeward vector (Collett, 1992). Landmark memory can guide bees to the hive even after artificial displacement of up to 11 km (Pahl et al., 2011). Another possible way to supplement the distance measurement is counting the landmarks passed on the way to a goal. Two studies have investigated this hypothesis so far.

Chittka and Geiger (1995) set out to test if bees can use a sequence of identical landmarks to estimate the distance to a goal. They set up a series of four yellow tents, and trained bees to forage at a feeder between the third and the fourth tent. A control experiment with empty feeders showed that the bees had learned to collect sugar water from this location, as only few bees landed on a distraction feeder on the way. By changing the number of tents in the setup, the experimenters created a contradiction between the perceived distance and the number of landmarks at which the bees expected the feeder. Would the bees search for the feeder at the learned distance, or after the learned number of landmarks? When the number of tents was increased to five (while keeping constant the distance of the last tent from the feeder), 74% of the bees landed at the feeder close to the trained distance, after passing four tents, and 26% of the bees landed at a shorter distance, at the position after the third tent. Increasing the number of landmarks to six caused most bees (58%) to land after tent four, a compromise between the trained distance and the number of landmarks passed during training. Thirty-three percent of the bees chose the feeder at the trained distance, after flying past five landmarks, and 8% of the bees landed after passing three tents. When the landmark arrangement was extended, so that the trained distance was reached after two landmarks, 78% of the bees chose the feeder at the training distance, and 22% flew 100 m further to a feeder after the third landmark. Thus, an increased density of landmarks led some bees to estimate the distance flown as being shorter, while a decreased landmark density led them to search at a greater distance. Chittka and Geiger (1995) concluded that the bees which did not land at the training distance must have had a representation of the number of landmarks to be passed between hive and food source, and referred to this behavior as “proto-counting.”

One criterion of true counting is the abstraction principle: the animal has to demonstrate the ability to use the number learned in one context in a transfer test on different objects (Gelman and Gallistel, 1978). As the landmarks were always yellow tents of identical size, this abstraction principle was not shown in this experiment. The bees’ numerical competence demonstrated here may be related to a serial memory for landmarks, since bees store stimuli along a route together with information about the next expected target (Collett and Kelber, 1988; Collett et al., 1993). Another possible explanation for the results is the change in optic flow caused by the tents on the way to the feeder. Since bees measure distance by the amount of movement over the retina, more tents on the way would cause them to underestimate the distance, while fewer tents would lead to an overestimation.

**Figure 1:**
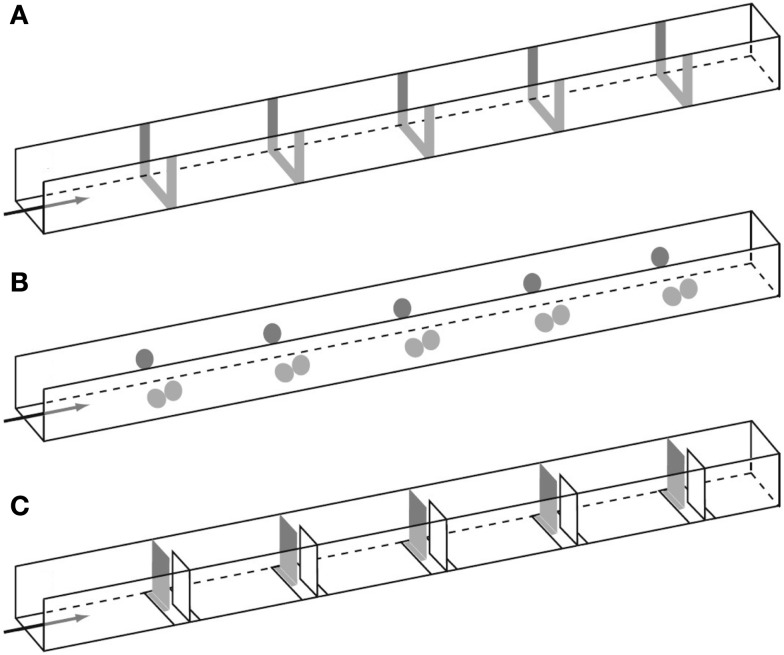
**Illustration of the experimental tunnels with landmarks consisting of (A) stripes, (B) circles, and (C) baffles spaced at regular intervals**. Adapted with permission from Dacke and Srinivasan (2008).

**Figure 2:**
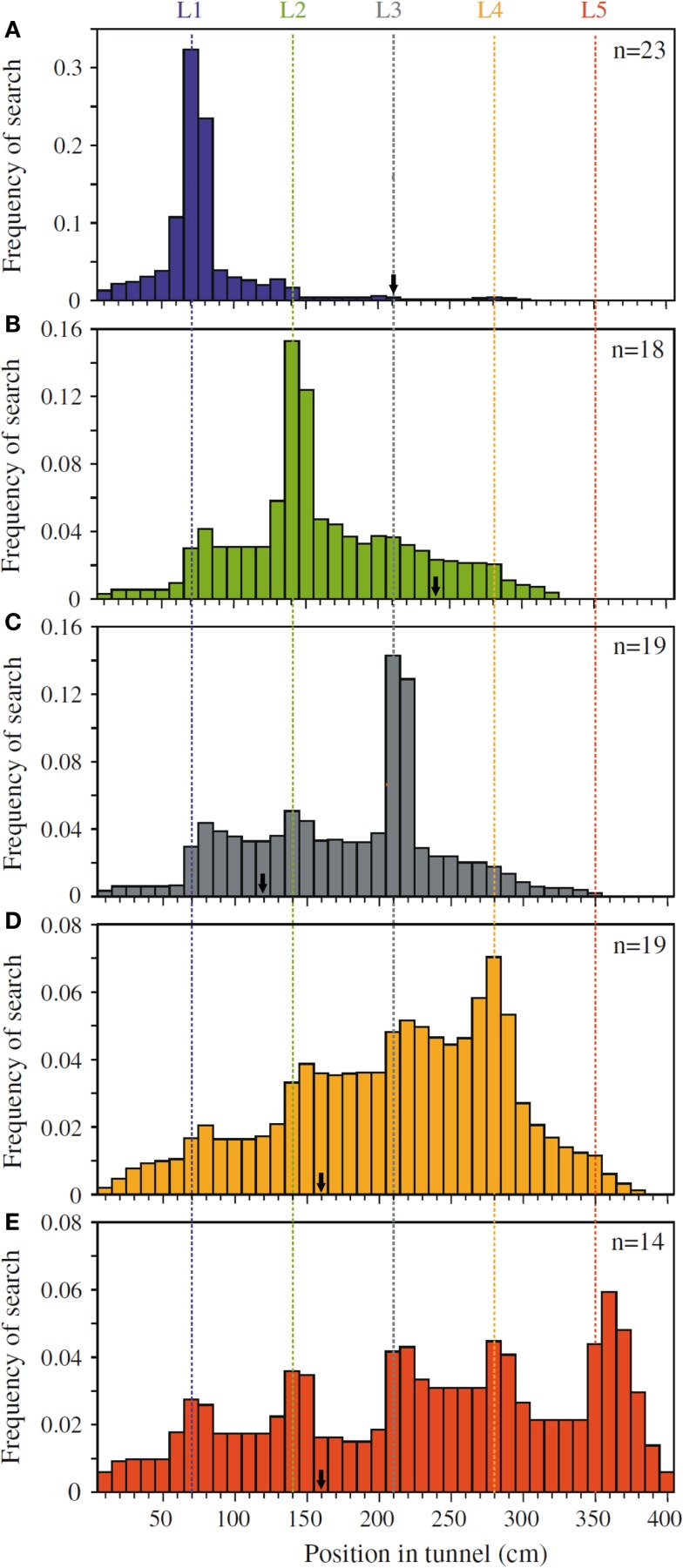
**Pinpointing the rewarded landmark in a series of landmarks**. Shown are the search distributions of bees that are tested after being trained to receive a reward at **(A)** landmark 1, **(B)** landmark 2, **(C)** landmark 3, **(D)** landmark 4, and **(E)** landmark 5. Bees trained to landmark 1 show a strong preference to search around landmark 1 **(A)**. Bees trained to landmark two similarly prefer to search near landmark 2 **(B)**, and so on **(C–E)**. The arrows mark the position of the rewarded landmark in the training, just prior to each test. Adapted with permission from Dacke and Srinivasan (2008).

Dacke and Srinivasan (2008)revisited the question of sequential counting in bees in a carefully controlled tunnel setup, in which the bees could not rely on odometric information to find the feeder. They trained bees to forage in a 4 m long and 20 cm wide tunnel containing five landmarks consisting of yellow stripes (Figure 1A). The position of the landmarks and the feeder was varied at 5 min intervals in order to prevent the bees from learning the feeder position based on its distance from the entrance. Different groups of bees were trained to find the feeder at landmark 1, 2, 3, 4, or 5, and their search distributions were measured. When the bees’ search behavior was at its best, the experimenters tested the bees in a new tunnel without reward, and recorded the bees’ search distribution. The animals trained to the first landmark searched mostly close to landmark 1 (Figure 2A), bees rewarded at the second landmark searched mostly around landmark 2 (Figure 2B), and so on (Figures 2C–E). Clear search peaks were only visible when the bees were trained to forage at landmarks 1–4. With an increasing number of landmarks to fly past, however, the search distribution became wider (Figure 2E). In the next experiment, Dacke and Srinivasan tested whether the bees could still identify the correct landmark in a different spatial layout. The animals were trained to collect sugar water from the third landmark as in Figure 1A, and then tested in one condition where the landmarks were closer together, and in a second condition where the landmarks were spaced irregularly. In both cases, the bees’ searches were centered on the third landmark. A third experiment was conducted to investigate whether the bees were using the number of landmarks, or the amount of yellow they passed on the way through the tunnel, to find the feeder. After training was conducted just as in Experiment 1, the animals were tested in a new tunnel without reward, and with yellow disks instead of stripes as landmarks (Figure 1B). With only 55% of area of the stripes, summing up the amount of yellow passed on the way would lead the bees to overshoot the feeder. The bees, however, showed a search pattern similar to the first experiment, with clear search peaks at the trained landmarks 1–4, and a wide distribution in case of landmark 5 (Dacke and Srinivasan, 2008). The bees were not summing up the amount of yellow, but were using the landmark number passed en route to locate the feeder. In a last experiment, the experimenters trained a group of bees in a tunnel with overlapping baffles as landmarks (Figure 1C). Unable to see from one baffle to the next, the bees were forced to count the landmarks in a truly sequential way in order to identify the correct one. After training the bees to forage at the third landmark, the test in an unrewarded tunnel showed that the animals still centered their search on the third landmark.

Dacke and Srinivasan showed that bees can keep track of a maximum of four landmarks passed en route. They also demonstrated that this behavior is not restricted to the type of landmark encountered in training, but can be applied to different landmarks in an abstract, object-independent way. Because the bees had a tendency to learn distance rather than number, the authors took pains in training the bees to disregard other information, leaving number as the only reliable cue to find the feeder.

In the featureless habitat of desert ants (*Cataglyphis fortis*), landmarks cannot help the animals to locate the nest entrance after a successful foraging trip. The ants constantly integrate distance and direction of their movement, enabling them to return home in a straight line from any position on their outbound path (path integration). The directional reference for the homeward vector is the polarization pattern in the sky, which the ants perceive in the specialized dorsal rim area of their compound eyes (Wehner, 2003). The mechanism by which distance is gaged was elucidated by Wittlinger et al. (2006). In a series of elegant experiments, they tested the hypothesis that *Cataglyphis* is using a “step counter” to measure travel distances. By elongating or shortening the ant’s legs (stilts and stumps), they caused the animals to overestimate (stilts) or underestimate (stumps) the travel distance, showing that the ants were really using the amount of steps taken to gage the traveled distance. The animals most likely do not literally “count” the number of steps, but integrate some parameter of leg movement during walking (Wittlinger et al., 2007). Further experiments are required to address the exact mechanism of the stride integrator. In contrast to desert ants, who use a discrete (countable) quantity to measure distance, honeybees use optic flow; a continuous (uncountable) quantity for distance measurement. Future studies should investigate the costs and benefits of discrete and continuous variables for distance measurement in different ecological contexts.

In 1996, Reznikova and Ryabko reported that red wood ants (*Formica* sp.) can assess the number of turns in a maze, and communicate this information to nest mates. They placed a scout ant near a sugar reward in a maze, where the ant could feed. The scout would then head home to the nest, and get in antennal contact with its group of foragers. After a timespan of antennal contact proportional to the number of turns in the maze, the foragers then headed out without the scout – and found the location of the feeder in the majority of trials. Olfactory cues were excluded by replacing the maze with a fresh, identical maze when the scout had returned to the nest. The authors conclude that the experimental animals estimate the number of objects passed along the way back to the nest to memorize the food location, and communicated this number to their nest mates. The foragers then used the communicated number to locate the correct branch on the maze (Reznikova and Ryabko, 1996, 2011). There is, however, no direct evidence that the number of branches was memorized by the scout, or received by the forager ant. Since ants can measure distance quite accurately, as shown in the study by Wittlinger et al. (2007), further experiments are required to exclude this possibility. The concept of symbolic communication in ants, however, is in itself extremely interesting, and should be investigated further.

## Number in Visual Discrimination

Since Karl Von Frisch’s initial visual discrimination experiments showed that honeybees can see colors (Von Frisch, 1914), the bee’s visual system has been investigated thoroughly in a large number of studies. Behavioral experiments on free-flying bees have played an important role in finding out about what a bee can see. Those studies revealed that bees can extract general identifying information from a stimulus, such as orientation (Van Hateren et al., 1990), radial symmetry (Horridge and Zhang, 1995), and bilateral symmetry (Horridge, 1996) including the orientation axis (Giurfa et al., 1996). Other characteristics of images such as color and size can be extracted and memorized as well (Horridge et al., 1992b; Ronacher, 1992).

One of the first studies on the visual use of number in insects was published in 1953 by Elmer E. Leppik. Inspired by the latest discoveries on the honeybees’ dance language (Von Frisch, 1951), he became interested in the question of whether, and how, insects could use numbers. Since many flowering plants which depend on insect pollination have a constant number of petals, he wanted to test whether pollinators had evolved a certain ability to discriminate blossoms based on the number of petals (Leppik, 1953). Despite the lack of controls and statistical tests, Leppik made some interesting observations. He found that the trained bees did well distinguishing between 1, 2, and 3, but had trouble discriminating between 3 and 4 petals. They remembered higher numbers only if these were expressed in symmetrical flower shapes. Since honeybees have an innate preference for symmetrical visual stimuli (Lehrer et al., 1995), the bees were probably using the overall shape of the flowers for the discrimination tasks involving numbers above 4. The limit to the bees’ numerical competence between 3 and 4, however, is similar to the findings in several recent studies reviewed here (Chittka and Geiger, 1995; Dacke and Srinivasan, 2008; Gross et al., 2009).

The studies by Chittka and Geiger (1995) and Dacke and Srinivasan (2008)have demonstrated that bees can keep track of the number of objects passed sequentially, i.e., one object at a time. The question remains, however, if bees can extract information about the number of simultaneously presented objects from a visual scene. Honeybees have been shown to generalize visual stimuli (Mazochin-Porshnyakov, 1969), and can learn concepts of “sameness” and “difference” in a delayed match-to-sample (DMTS) task (Giurfa et al., 2001). In order to investigate this question, Gross et al. (2009) trained bees in the DMTS paradigm to make decisions about the sameness or difference of visual stimuli based on the number of objects present in a stimulus.

**Figure 3:**
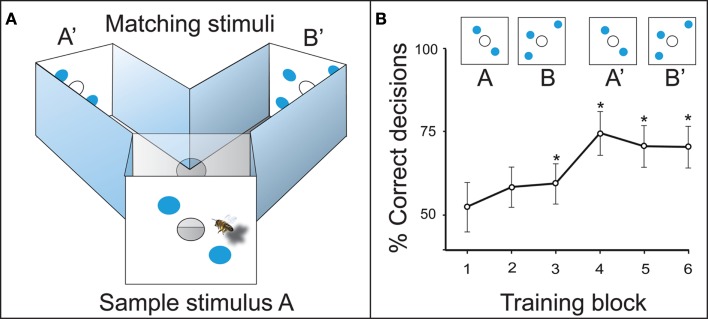
**(A)** Delayed match-to-sample (DMTS) setup in a Y-maze. The bee has to memorize sample stimulus A, and compare it to the matching stimuli A’ and B’ inside the maze. A’ leads to the reward in this example. **(B)** Learning curve of the DMTS experiment. *Denotes a preference for the matching stimulus significantly different from random choice.

In the initial training, the experimental bees learned to fly into a Y-maze (Figure 3A). The animals had to memorize the sample stimulus at the maze entrance, recall it inside the decision chamber, and choose the matching stimulus to get a sugar reward. When the two-dot stimulus A was the sample at the maze entrance, the bees were rewarded for choosing the two-dot matching stimulus A’ inside the maze. When the three-dot sample B was presented, they had to choose the three-dot matching stimulus B’ to collect their sugar reward (Figure 3A). The animals learned to solve this task with a precision of 70–75% after three to four training blocks, which is 20–30 visits per individual bee (Figure 3B). This relatively simple task could be solved in a number of ways which do not require counting, i.e., by image matching, adding combined area or edges, or by matching the illusory contours formed by the objects (Horridge et al., 1992a). Therefore, a number of experiments were designed to exclude other information – so that the number of elements was the only reliable cue for the bees.

**Figure 4:**
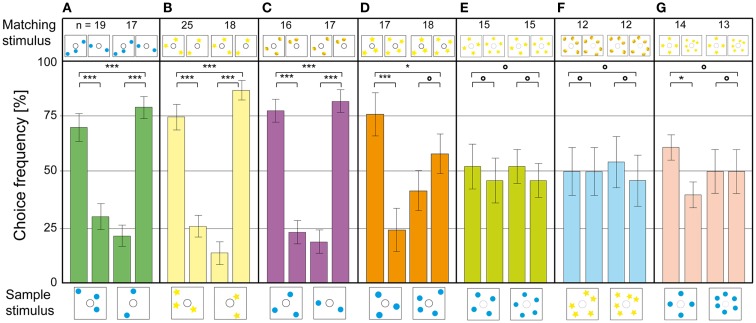
**Number-based decision-making in a delayed match-to-sample task**. The stimulus below each pair of bars is the sample, and that above each bar is the respective matching stimulus. The data represent the pooled first choices of individual bees. **(A)** The configuration of dots on the sample and matching stimuli is randomized. **(B)** The blue dots are replaced by yellow stars, to test for abstraction to unknown stimuli. **(C)** Sample and matching stimuli are composed of different elements. **(D–G)** Bees trained to discriminate between 2 and 3 are tested on stimuli with **(D)** 3 and 4 elements, **(E)** 4 and 5 elements, **(F)** 5 and 6 elements, **(G)** 4 and 6 elements. *n* Denotes number of bees per condition. Error bars show standard error. ***Denotes statistically significant difference at *p* < 0.001, **denotes *p* < 0.01, *denotes *p* < 0.05, and O denotes *p* > 0.05. Modified from Gross et al. (2009).

**Figure 5:**
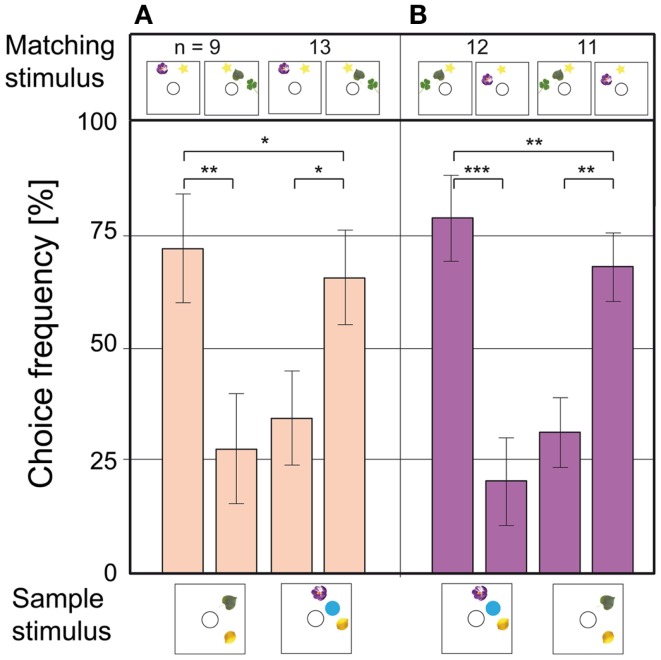
**Control test for illusory contours and misdirecting cues**. The objects are arranged in lines of equal length to prevent the formation of illusory contours. In each configuration, only one object appears both in the sample and in the matching stimuli as “misdirecting” cue. **(A,B)** Misdirecting cues are the green leaf in the 2 to 2 match and the purple flower in the 3 to 3 match. Notations used here are the same as in Figure 4. Modified from Gross et al. (2009).

As a first step, the bees were presented stimuli where the positions of the blue dots had been randomized. The animals solved this without any additional training, showing that they did not use image matching to find the reward (Figure 4A). In the next experiment, the blue dots were exchanged for yellow stars; new objects which the bees had never encountered during training. They transferred the matching rule to the new objects without decreasing choice performance (Figure 4B). In the next step, the sample stimulus consisted of blue dots – while the matching stimuli contained yellow lemons. The bees solved this task as well, with a high accuracy of around 80% (Figure 4C). These results show clearly that the bees were applying the learned rule in an abstract, object-independent way, which is one criterion for true counting (Gelman and Gallistel, 1978). In two further experiments, the blue dot stimuli were controlled for edge length and combined area, which did not decrease the frequency of correct choices (Gross et al., 2009). Thus, the bees were not using spatial frequency or area summation to choose the rewarded stimulus. When a new number of objects was introduced (3 vs. 4 blue dots), the animals had no trouble solving the 3 to 3 match. In the 4 to 4 match, however, the choice frequency dropped to chance level (Figure 4D). In another experiment with similar objects as sample and choice stimuli (yellow lemons and yellow stars), the bees could solve the four to four match as well (Gross et al., 2009). Thus, the limit of the bees’ number discrimination ability seems to be between 3 and 4. When they were tested on configurations with 4 vs. 5 (Figure 4E) or 5 vs. 6 objects (Figure 4F), the choice distribution dropped to chance level. Interestingly, when the bees were tested on 4 vs. 6 objects, they could do the 4 to 4 match, but failed to do the 6 to 6 match. The honeybee’s sense of number does not follow Weber’s law, which states that the just-noticeable difference between two stimuli is proportional to the magnitude of the stimuli. If the bees were making relative numerousness judgments, a 2 vs. 3 discrimination would mean that they should be able to discriminate 4 vs. 6 as well. The fact that this is not the case indicates that the bees were using absolute number, instead of relative quantity, to identify the matching stimulus. These results are similar to those obtained for 22 weeks old human infants in a habituation – dishabituation procedure. The infants noticed a difference between arrays of 2 and 3 objects, but not between arrays of 4 and 6 objects (Starkey and Cooper, 1980).

Honeybees can perceive illusory contours formed by elements on a visual stimulus (Horridge et al., 1992a). Since two objects always form a line, and three objects tend to form a triangle, additional experiments were carried out to control for those lower order cues. The objects were arranged in straight lines of equal length, to prevent the bees from using the overall shape of the elements for the matching task (Figures 5A,B). Additionally, the stimuli were made as dissimilar as possible, by using different objects in sample and choice stimuli. An attempt was made to guide the bees deliberately to the wrong stimulus: in the 2 to 2 matches in Figures 5A,B, the green leaf is the only object present in the sample and in a choice stimulus; the other objects are all unique. If the bees were to match the stimuli based on individual objects, the green leaf would guide them to the wrong side of the maze. In the 3 to 3 match in Figures 5A,B, the purple flower serves the same purpose. As the data in Figures 5A,B show, the choice performance in this experiment was still high. Even this deliberate effort did not fool the bees: they clearly based their decisions on the number of objects present in the stimuli.

## Number in Foraging Strategies

Bees can visit hundreds of flowers on one of their excessive foraging flights. Since the nectar content cannot be judged from a distance, the animals have evolved a number of strategies in order to prevent revisiting previously depleted flowers. When foraging across several patches of flowers, bumblebees use fixed foraging routes between the patches. This increases foraging success when competing with other pollinators [reviewed in Ohashi and Thomson (2009)]. The sequence of flower visits is at first learned in the order of flower discovery – and subsequently optimized for shortest flight distance (Lihoreau et al., 2010, 2011). When foraging on inflorescences, bumblebees tend to visit the flowers sequentially, from the bottom to the top (Pyke, 1979; Waddington and Heinrich, 1979). Honeybees mark visited flowers with a repellent scent – and reject recently visited flowers (Giurfa and Nunez, 1992; Giurfa, 1993).

In 2011, Bar-Shai et al. proposed that the number of nectaries visited per flower could be used as an information source to prevent revisits of depleted nectaries, as long as the number of nectaries is constant. If the animals were using number as a departure cue, they should flexibly adapt their departure strategy to the number of available food sources. The authors tested this hypothesis in field observations and lab experiments. Bumblebees naturally collect nectar from *Alcea* *setosa* flowers, which offer a constant number of five nectaries (Bar-Shai et al., 2011b). Observing foraging bees in the field, the authors found that the bees most commonly departed after having probed the five nectaries (92% of visits). Revisits of depleted nectaries happened rarely, in only 1.1% of the cases. In order to test whether the bees could adapt their foraging strategy to a different number of available food sources per patch, the authors trained bumblebees to forage at artificial feeders, set up in two patches with three feeders each. Only two rewards could be accessed per patch. After probing two feeders, the remaining one was closed so that the bee had to visit the other patch to gather more sugar water, or return to the hive. The bees adjusted their frequency of patch probings before departure, and the probability of leaving a patch after receiving the second reward significantly increased during the experiment. Non-numerical flower departure cues, such as ingested nectar volume, time spent on flower, spatial attributes of the flowers, and scent marks on the flowers could be excluded. It took the bees exceptionally long to learn to leave a patch after two rewards, indicating that number is more difficult cue to learn than color, size, or scent. The study shows that bumblebees can learn to leave a feeding location after receiving a fixed number of rewards. This involves sequential tagging of items in a fixed order (ordination), and using the last tag to determine number of items (cardination); two of the underlying basic principles of numerical ability (Gallistel and Gelman, 2005).

The authors conclude that bumblebees can use numerical regularities in food distribution to enhance their foraging efficiency, and that this may provide the selective drive in the evolution of numerical competence (Bar-Shai et al., 2011b). In order to investigate this hypothesis, the authors observed more primitive, solitary *Eucera* sp. bees that forage on the same *A. setosa* flowers for comparison (Bar-Shai et al., 2011a). In the solitary bees, flower departure after probing five nectaries was less common (26%) than in bumblebees (48%), and the likelihood of revisiting a nectary already depleted by the same individual was higher in *Eucera* sp. (7.8%) than in bumblebees (1.1%). The solitary bees also displayed “inspection turns,” where they approached a nectary, but encountered a scent mark and turned back. When these cases are taken out of the analysis, departures after six probings become more likely than departure after five probings. Measuring the duration of each inspection, the authors found that the last inspection before departure was usually the shortest. Time spent on a flower, departure after ingesting a certain volume of nectar and spatial characteristics of the flower could be excluded as departure clues. Most likely, the bees were using a reward-based patch departure rule, assisted by scent marks: whenever a nectary is empty, or carries a scent mark, depart, and visit the next flower. Non-numerical cues are the most parsimonious explanation for the results, but the use of number by solitary *Eucera* sp. bees cannot be ruled out. If they do possess a form of numerical competence, it is less exact than in bumblebees (Bar-Shai et al., 2011a,b).

## Conclusions

The papers reviewed above have shown that numerical competence in insects is a worthwhile topic of investigation, with a variety of experimental approaches being available to test the limits of these animal’s abilities. The fact that a range of very different behavioral assays has indicated the number 4 to be the upper limit of insect numerical competence, strongly suggests that this is a key cognitive constraint that requires detailed and rigorous study. The same limit was found when stimuli were encountered sequentially (Chittka and Geiger, 1995; Dacke and Srinivasan, 2008; Bar-Shai et al., 2011a,b) as well as simultaneously (Leppik, 1953; Gross et al., 2009; Gross, 2011). Other questions raised by the above studies that need to be properly investigated include an elucidation of the exact mechanism(s) by which insects discriminate between these small numbers, as well as the interactions, if any, of numerical discrimination with other cognitive capacities, such as a time sense. Vertebrate studies have revealed striking parallels between these two faculties, especially for smaller values [i.e., small numbers and short time intervals; Buhusi and Cordes (2011)]. Since bumblebees can be trained to learn specific time intervals (Boisvert and Sherry, 2006), these insects, along with honeybees, appear to be an ideal model organism in which to study the neural correlates of both the numerical and interval timing abilities, as well as the commonalities between the two systems.

In summary, the studies presented in this review reveal the great potential of insects to inform current theories of numerical perception and competence. Given the complex nature of this cognitive domain, however, future studies should address often-neglected variables such as age and experience, and individual differences (Dyer, 2012) to arrive at a more accurate and comprehensive picture of numerical ability. Fortunately, these are variables that, in social insects, can be manipulated with some effort, so as to produce better-controlled experimental protocols. The effects of attention-like processes, as have been seen in honeybees (Giurfa, 2004) and in *Drosophila* (Van Swinderen and Flores, 2007), also have the potential to indicate more precisely the mechanism by which the former group of insects is able to discriminate between small numbers. Is attention more important in maze studies, such as that of Gross et al. (2009), where the bees are allowed to examine the visual stimuli for an extended period of time, before making a decision? Or might attention play a greater role in studies such as that of Chittka and Geiger, where the bees need to extract the information relevant to a landmark from a noisy background – as she flies past it en route to a feeder? Finally, the recent proposal that bees may possess different visual systems for pattern discrimination at close range vs. at a distance (Dyer and Griffiths, 2012) may also have a bearing on the counting procedure being used by individual foragers. The experiments described in this review required bees to either estimate number by, e.g., flying very close to a visual pattern, or from a distance, by, e.g., flying past a prominent landmark, and it is possible that different mechanisms are employed by the bees in these two scenarios.

“Number” is a primary visual feature of a scene, along with color, contrast, size and speed (Burr and Ross, 2008), and many animals have evolved the ability to make use of this information. The visual recognition of small numbers of items could be achieved during early sensory processing, and the same may be the case for olfactory and auditory “scenes.” In order to understand the complexity of numerical cognition, a bottom-up approach investigating the neural circuitry required for number recognition is necessary (Chittka et al., 2012).

## Conflict of Interest Statement

The authors declare that the research was conducted in the absence of any commercial or financial relationships that could be construed as a potential conflict of interest.
